# Public Health Hackathon: empowering high school students as tomorrow’s leaders and innovators in public health

**DOI:** 10.3389/fpubh.2026.1745900

**Published:** 2026-02-11

**Authors:** Ashish Joshi, Niharika Jha, Michelle Jeu, Kami Geron, Lori Ward

**Affiliations:** School of Public Health, University of Memphis, Memphis, TN, United States

**Keywords:** hackathon, high school students, innovation, leadership, public health, Public Health Hackathon

## Abstract

Preparing the next generation of public health leaders requires innovative educational strategies that foster creativity, collaboration, and practical problem-solving skills. Hackathons—short, intensive innovation challenges—provide a structured platform for students to co-create solutions to real-world problems. The University of Memphis School of Public Health launched the “RE-AIM Public Health IDEAS through the Lens of Youth Hackathon” series to empower high school, undergraduate, and graduate students to engage in public health innovation using design thinking and a human-centered approach. The *Public Health Hackathon* applies the SMAART model combined with principles of human-centered design and design thinking to guide participants through ideation, prototyping, and solution development. The 3rd annual hackathon, held in 2024–2025, involved 87 participants from 9 institutions across four countries (United States, India, Malta, and Uganda). Through workshops, mentorship, and structured innovation sessions, students developed public health campaigns and prototypes addressing locally and globally relevant challenges. Participants demonstrated increased awareness of public health careers, improved communication and teamwork skills, and enhanced understanding of health promotion and community engagement strategies. The program was implemented as an extended design-competition format with optional virtual workshops (problem identification and research; ideation and storyboarding; prototyping and implementation planning; pitching and communication), centralized technical assistance, and rubric-based evaluation of abstracts and pitches by an interdisciplinary panel of experts. Student teams developed diverse public health project concepts, including campaigns and prototypes, addressing locally and globally relevant health challenges. This manuscript describes the Hackathon’s design, implementation, and evaluation approach and highlights its potential as a pragmatic model for early public health workforce exposure and youth-led community health innovation. The RE-AIM Public Health IDEAS Hackathon model effectively bridges public health education and workforce development by empowering future leaders as agents of change and innovation within their communities.

## Introduction

Hackathons provide an opportunity for innovators to brainstorm creative ideas to solve a defined problem. Traditionally, hackathons have served as a methodology to foster the rapid generation of innovative solutions leveraged by digital technologies and immersive collaboration across a broad range of multidisciplinary contexts (1). In educational settings, hackathons are transformative tools for engaging students, enhancing knowledge retention, and developing both soft and technical skills. Importantly, hackathons offer students a hands-on, experiential learning opportunity to address broad systemic issues that fall beyond the remit of regular educational curricula (2). In recent times, the application of hackathons has expanded notably into sustainable health innovation, with events designed to bring together diverse stakeholders and address real health needs. For example, a health hackathon in Berlin directly involved patients and healthcare professionals in all stages of the innovation process, demonstrating a user-centered and transdisciplinary approach to developing digital health solutions and producing systematic recommendations for future innovation events (3). Similarly, rehabilitation-focused hackathons have been documented in the medical literature as interdisciplinary events that unite clinicians, engineers, designers, and others to rapidly prototype healthcare solutions while fostering cross-sector collaboration (4). Beyond academic case studies, recent applied events such as the ReFlow: Menstrual Health Innovation Hackathon in India have mobilized students, startups, NGOs, and other community partners to co-create sustainable solutions for menstrual health challenges, highlighting the broader *real-world impact* of health-oriented hackathons (5).

Peer-based learning is a well-established pedagogical approach in public health education, grounded in social learning theory and constructivist models of education, which emphasize learning as a collaborative, socially mediated process (6). Through peer interaction, learners actively construct knowledge, reinforce understanding, and develop critical thinking and communication skills essential for public health practice. Within workforce development frameworks, peer learning has been shown to strengthen professional identity formation, teamwork, leadership, and problem-solving competencies—skills that are increasingly recognized as core to the future public health workforce (7). The Health Hackathon empowers students to become agents of change within their school communities while inspiring them to explore careers in the health workforce. School-based preventative health interventions targeting adolescents can significantly enhance health literacy and foster health behaviors that persist into adulthood (8). Peer education is a well-established approach for delivering such interventions, with evidence demonstrating positive impacts on students’ knowledge, attitudes and behaviors across various health topics (9). However, to ensure effectiveness and avoid unintended harm, peer leaders require close support from adults to guide their responsibilities and communicate accurate health messaging (10).

Traditionally, hackathons are intensive 1–2 day events emphasizing rapid prototyping and pitching. In this format, teams work under tight time constraints to produce working prototypes or solution demos by the end of the event, supporting fast learning and innovation in health and technology contexts. For example, health-focused hackathons have been documented as short, interdisciplinary events that bring together participants from diverse backgrounds to collaboratively design and pitch healthcare solutions within a discrete time frame (11). In contrast to this time-bounded hackathon model, design competitions and similar student challenge formats often span weeks to months, providing extended periods for structured workshops, mentorship, ideation, research, iterative prototyping, and reflective refinement, which are aligned with deeper learning outcomes and real-world problem solving. For instance, global case competitions in public and global health challenge student teams to work over several weeks to develop thoroughly researched proposals and multimedia deliverables around pressing health issues, fostering critical thinking, teamwork, and sustained engagement with complex material (12). Recognizing these differences, the School of Public Health, University of Memphis adopted an extended design competition format to better serve high school students’ developmental needs. This extended timeframe allowed students to build *foundational public health knowledge*, receive ongoing expert guidance, engage meaningfully with mentors, and develop critical workforce skills in a balanced way alongside their academic commitments—thereby prioritizing educational depth over the rapid pace typical of traditional hackathons.

The design competition hackathon methodology offers an innovative solution to these challenges by creating a structured and supportive environment where students can collaborate with public health experts to develop and test their ideas. Leveraging this methodology and youth voice to foster impactful health innovation, the University of Memphis School of Public Health launched the innovative *Public Health Hackathon* series “RE-AIM Public Health IDEAS through the Lens of Youth Hackathon”, first of its kind initiative to engage high school students to design, develop and implement out of box, creative ideas following human-centered approach and design thinking principles to address public health challenges that impact in the local or global settings. This initiative seeks to create public health awareness among high school students and guides students to develop a health promotion campaign for their school community, their families and the communities they live in. It further engages the students broadly with public health experts across various sectors including health department, hospitals, insurance organizations, pharmacy, start up and community organizations and broaden awareness of careers in the field of public health.

The initiative “RE-AIM Public Health IDEAS through the Lens of Youth Hackathon”*, public health hackathon* is driven by young people, their innovation, creativity and their will to be drivers in the delivery of public health approaches towards advancing Sustainable Development Goal three, good health and wellbeing.

## Objectives

The objectives of the *public health hackathon* include (i) to sensitize individuals about the field of public health (ii) assist future public health leaders to come up with out-of-box solutions to address public health challenges of the 21st century, (iii) connect, collaborate, and coordinate with students from across the world to build teams to solve common public health challenges, and (iv) to create a platform that enables future leaders to bring together ideas on how today’s pressing public health problems can be addressed across various local and global settings.

**Figure 1:**
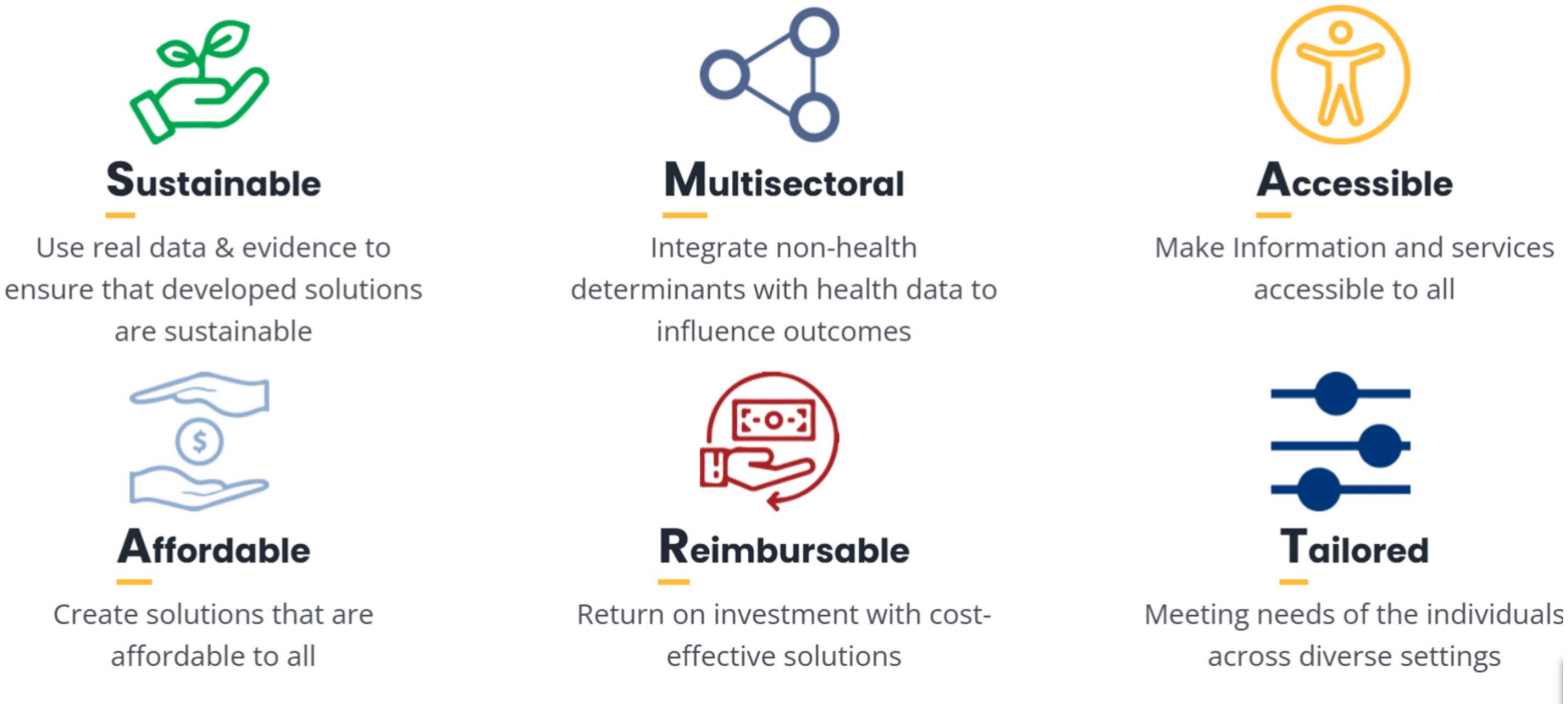
SMAART model.

**Figure 2:**
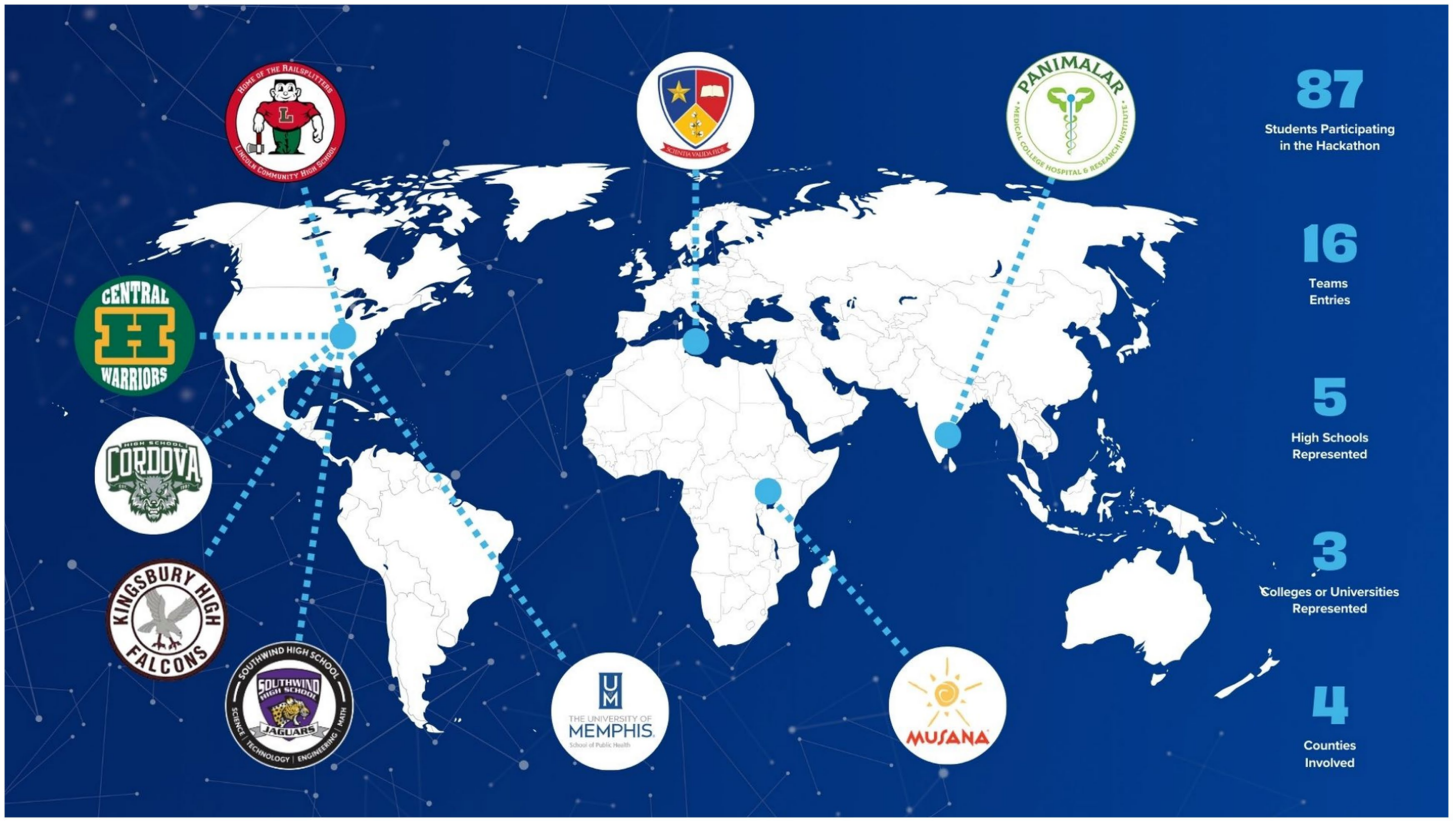
Geographic distribution of participants of the 3rd Public Health Hackathon.

This first of its kind initiative, facilitates teamwork, critical thinking and problem-solving skills and challenge high students to present creative and innovative solutions to address public health challenges both in local and global context and contribute towards the improvement of good health and wellbeing of communities across geographic settings (Figures 1, 2).

### Conceptual framework

The *Public Health Hackathon* is developed using the combined principles of human-centered Design approach and design thinking approach using the SMAART model (13). SMAART model is based on a human-centric & impact-driven approach with the goal of enhancing the health & wellbeing of communities locally and globally. The SMAART Model is created to solve real problems with real solutions driven by data and evidence to design and develop solutions that are Sustainable, Multi-sectoral integrating non-health determinants with health data to influence outcomes, make information and services Accessible to all, create solutions that are Affordable to all, Reimbursable meaning there is a return on investment with cost-effective solutions and Tailored to meet needs of the individuals across settings.

## Methods

- Initiative overview:

◦ The RE-AIM Public Health IDEAS Hackathon is a global, hybrid innovation program designed to engage high school and university students in developing youth-led, research-informed public health solutions aligned with Sustainable Development Goal 3 (SDG 3): ensure healthy lives and promote wellbeing for all ages. The Hackathon integrates principles of public health education, human-centered design, and experiential learning to strengthen early workforce pipelines and foster youth leadership in public health innovation.

- Initiative launch:

◦ RE-AIM Public Health IDEAS through the Lens of Youth Hackathon is an innovative initiative launched in August 2022. This initiative is aimed at instilling Leadership and Educational Advancement Among students to Solve Public Health Challenges of the 21st Century (LEAP). RE-AIM Public Health IDEAS Hackathon framework was used to implement Model of Public Health Education in High schools through a range of initiatives including Public Health Dual Enrollment, (b) Public Health Clubs in High Schools and (c) Public Health Hackathon. In our inaugural launch of the *public health hackathon*, a few selected high schools were reached out to participate in the hackathon with an opportunity to generate solution-based ideas. As we connect with future workforce locally, nationally, and globally, we are beginning to embark on solving public health challenges on a greater scale.

- Participation outreach:

◦ Institutions engaged with University of Memphis School of Public Health high school initiative RE-AIM Public Health IDEAS through the Lens of Youth Hackathon or those having collaborations with the University of Memphis School of public health were initially reached out to participate in the *public health hackathon*.

- Target audience: The platform gave an opportunity to high school students (grade 9–12), and undergraduate and graduate public health students enrolled at the University of Memphis School of Public Health.
- Why participate: This initiative allows the participants to achieve their objectives and turn ideas into reality. It offers the opportunity to solve public health problems through innovative and sustainable solutions. These solutions contribute to the improvement of health and wellbeing. The participants become champions of public health change.

Brief about the *Public Health Hackathon*: The Public Health Hackathon engages students in developing creative, solution-oriented responses to pressing public health challenges across local and global contexts. Participants competed individually or in teams, with a defined theme for each annual Hackathon held during 2022–2023, 2023–2024, and 2024–2025. The inaugural Hackathon (2022–2023) involved students from three high schools across two countries (the United States and India). The second Hackathon (2023–2024) expanded participation to include both high school and university students, engaging institutions in the United States and Portugal, with eight student teams participating. The third Hackathon (2024–2025) further scaled the initiative to include 16 teams and 87 participants from four countries—the United States, India, Malta, and Uganda. Implementation process included four major steps:

- Problem identification: pinpoint critical public health issues

◦ The workshop guided students through the design process by helping them clearly define the problem. Students were prompted to consider who is affected, how they are affected, and the scale and scope of the issue. They learned how to define and frame a problem as the foundation for solution design, while also thinking critically about the stakeholders involved and how to use data collection and research to inform their solutions.

- Idea generation: develop creative and feasible solutions

◦ The concept of ideation was introduced to help students generate a wide range of ideas using techniques such as structured brainstorming, mind mapping, “How might we” statements, and storyboarding. The workshop provided a framework for exploring diverse perspectives, considering all possible solutions, and beginning to plan for project creation and execution.

- Project design: turn ideas into actionable projects

◦ The workshop guided students in transforming solution ideas into real, actionable projects. Using tools such as prototyping, wireframing, and iterative refinement, they learned how to test and improve their concepts with feedback. This stage emphasized both creativity and planning. Students were encouraged to collaborate with stakeholders, develop project plans and roadmaps, and pilot their ideas to explore what works in practice and ensure meaningful public health impact.

- Pitching: refine your presentation skills and pitch your ideas to an expert panel

The workshop prepared students to effectively present their solutions to an expert panel. They learned strategies for showcasing their ideas, gaining buy-in, and communicating their work in ways that engage community members, generate interest, and highlight the impact of their projects.

### Details of 3rd *Public Health Hackathon*

#### Who were the participants?

Eighty-seven individuals from four countries including United States, India, Malta and Uganda participated in the 3rd annual hackathon. The participants were from high schools (grade 9–12), and College and University settings. High schools in the US included Memphis Shelby County Schools Cordova, Central, Kingsbury, and Southwind, Lincoln Community in Chicago, and De La Salle College Sixth Form School (Malta), University of Memphis School of Public Health (USA), Panimalar Medical College (India), and Musana Vocational (Uganda). Sixteen teams participated in this hackathon with 13 teams submitting a complete abstract and pitch while 3 teams only submitted an abstract.

#### Key milestones of Public Health Hackathon

The 3rd University of Memphis School of Public Health Hackathon was announced on September 9, 2024. Participants primarily included high schools with established UofM SPH Public Health IDEAS Clubs, along with partner schools and institutions in Uganda, Malta, the United States, and India. Design thinking and human-centered design workshops were conducted in January and February 2025 for interested participants. Abstracts were due February 10, 2025, with final project pitches submitted in April 2025 (Supplementary Table S1).

#### Participation outreach and recruitment for the 3rd annual hackathon (2024–2025)

Participation outreach utilized a multi-channel recruitment strategy targeting high school, university, and international audiences. Recruitment leveraged existing IDEAS Club networks through Memphis–Shelby County Schools Career and Technical Education (CTE) partnerships, along with direct outreach to school administrators, collaboration with Public Health Club advisors, and dissemination through partner organizations, including the iEARN global education consortium.

Additional outreach included social media promotion, email campaigns to University of Memphis School of Public Health and dual-enrollment students, campus flyers, and a dedicated Hackathon webpage with registration materials. Two virtual informational sessions were hosted. Schools were contacted beginning in September–October of the academic year. Participation was free, with certificates provided to all abstract submitters and additional recognition awarded to selected teams.

#### Participants and selection procedures (2024–2025)

Participants included high school students (grades 9–12), university students, and invited public health professionals serving as judges. High school participants were recruited through established Public Health IDEAS Clubs, class-based participation, or voluntary enrollment, depending on school context. University students self-selected to participate through open outreach and institutional communication channels.

Judges were invited by the Dean of the School of Public Health and represented public health practice, academia, policy, and industry. Participation expectations emphasized project design and conceptual development rather than full implementation, with evaluation criteria focused on innovation, feasibility, and public health impact.

#### Hackathon design and timeline (2024–2025)

The Hackathon followed a phased, overlapping implementation model rather than a strictly linear sequence. Recruitment, informational sessions, workshops, mentorship, and abstract development occurred concurrently. Workshops were offered between January and February and overlapped with abstract submission and screening. Mentorship and technical assistance were available throughout this period. Following abstract review, selected teams advanced to pitch development and final judging. The Hackathon culminated in a hybrid awards ceremony.

#### Implementation requirements (2024–2025)

Each participant must submit initially an abstract, limited to 500 words, that describes the public health problem that they plan to address, the innovative solution that is proposed, the approach they plan to take to implement, ways they plan to measure impact and how they plan to address sustainability of the solution that is suggested. The abstracts are then reviewed by an independent panel of experts from varied backgrounds representing members of the civic society, policymakers, academicians, entrepreneurs, academicians, researchers, and public health practitioners. The participants are then introduced to the resources and workshops based on human-centered design principles. Participants then submit a 1–3-min YouTube video pitch to present their proposed idea. The 6-point judging criteria “CREATE” evaluates hackathon submissions based on their Creativity, Research-based, Entrepreneurial approach, Accessible Transformative and Effective solution.

#### Workshop curriculum and instructional design

A structured series of four capacity-building workshops supported participant learning and project development. Workshops were delivered virtually and recorded to ensure equitable access for local and international participants. Attendance was optional but strongly encouraged, and recordings were housed on the Hackathon website for asynchronous reference.

Workshop 1: Problem identification and research introduced students to public health problem framing using design thinking and epidemiologic principles. Instruction emphasized research question development using the FINER and PICOT frameworks, stakeholder identification, ethical considerations, and use of credible public health data sources (e.g., CDC, WHO, Healthy People 2030, local health department data).

Workshop 2: Ideation and storyboarding focused on translating research-informed problems into potential solutions using human-centered design tools. Techniques included brainstorming, mind mapping, empathy mapping, “How Might We” questions, journey mapping, dot voting, and storyboarding. Students were encouraged to generate multiple ideas before narrowing concepts based on desirability, feasibility, and viability.

Workshop 3: Project design, prototyping, and implementation planning guided participants in transforming ideas into tangible project designs. Instruction included low-fidelity prototyping, wireframing, role prototyping, service prototyping, feedback collection, and iterative refinement, with attention to implementation constraints, sustainability, and scale.

Workshop 4: Pitching and communication prepared participants to communicate their projects effectively to interdisciplinary audiences. Content aligned explicitly with evaluation rubrics and emphasized abstract-to-pitch translation, evidence-based storytelling, visual communication, elevator pitching, and ethical information sourcing.

Students worked individually or in teams depending on context and access to local facilitation. Participants affiliated with established Public Health IDEAS Clubs received in-person support, while others received virtual technical assistance and feedback by appointment. Instructional templates and examples were provided throughout.

A summary of workshops, tools, and expected outputs is provided in Supplementary Table S2.

#### Mentorship and technical assistance

Mentorship followed a centralized facilitation model led by the Project Manager for Strategic Initiatives (MJ). This role provided process facilitation, design guidance, public health framing, and feasibility feedback across the Hackathon cycle. Support was delivered in person for schools with Public Health IDEAS Clubs and virtually for other participants through email consultation and scheduled appointments. While mentors did not undergo formal training, the adaptive facilitation model allowed for responsive, individualized guidance aligned with student needs and project maturity.

#### Evaluation and rubric development

Evaluation rubrics for abstracts and pitches were developed *de novo* by program leadership and reviewed by faculty leadership prior to implementation. Rubrics were shared with participants following workshops and before abstract submission. Abstracts were reviewed in a blinded manner by an interdisciplinary panel of judges using standardized criteria assessing problem definition, innovation, feasibility, impact, and sustainability.

Advancement from abstract to pitch was determined by rubric-based scoring, with separate consideration for high school and university submissions. Full evaluation rubrics and scoring criteria are provided in Supplementary Tables S2, S3.

#### Ethical considerations and youth voice

Youth voice was positioned as a core component of the Hackathon, framed within responsibilities of ethical communication and evidence-based public health practice. Workshops addressed misinformation, responsible data sourcing, and the limitations of social media and generative AI tools. Participants were encouraged to critically evaluate information sources and cite data appropriately; however, no formal enforcement mechanisms beyond rubric-based evaluation and mentor feedback were implemented.

#### Judging and evaluation

Each team submitted (i) A written abstract (scored out of 50 points), (ii) A 3-min recorded pitch, and (iii) A 2-min live Q&A session with the judging panel (pitch scored out of 80 points). Abstracts were evaluated using the criteria CREATE meaning that the solutions proposed should be (a) Creative, (b) Research-oriented, (c) Entrepreneurial, (d) Accessible, (e) Transformative, and (f) Effective. Simultaneously pitch evaluation criteria highlighted the importance of presentation being Innovative and Creativity, Relevance and Impact, Feasibility and Implementation, quality of presentation, Evidence Use and Data Support, Scalability and Sustainability and Team Collaboration and Passion.

## Results

A total of 87 students from 9 institutions across 4 countries (United States, India, Malta, and Uganda) participated in the 2024–2025 RE-AIM Public Health IDEAS Hackathon. Six high schools and three universities/colleges were represented, forming 16 teams, of which 13 submitted a complete abstract and pitch and 3 submitted an abstract only.

The hackathon, held on May 5, 2025, was preceded by four virtual workshops (problem framing, ideation, project design, pitching) and 5 months of mentorship, technical support, and office hours. Students developed 16 projects addressing sexual health, obesity prevention, mental health, substance abuse, oral health, and environmental sustainability (Supplementary Table S4).

Projects were evaluated by a multidisciplinary judging panel (public health, technology, academia, and finance). Awards recognized innovation, feasibility, clarity, and impact. There were teams that were honorable mentions in the innovation and the impact categories.

The combination of workshops, mentorship, and pitching equipped participants with practical skills in design thinking, communication, and teamwork, aligning with workforce development goals. The hackathon functioned not only as a competition but also as an experiential learning environment supporting workforce-relevant skill development. In total, 66 local students across 11 Memphis-based teams and international peers from Malta, India, and Uganda contributed. Projects highlighted the integration of digital tools, community-based approaches, and sustainability-focused solutions. The hackathon concluded with an awards ceremony on May 5, 2025, celebrating student-led innovation in public health.

### Limitations

Participation in workshops was optional and engagement levels varied across teams. Outcomes were assessed using project submissions and rubric-based evaluations rather than longitudinal measures of skill acquisition or career impact. Findings reflect a pragmatic, applied implementation and may not be generalizable to all educational contexts.

## Discussion

This study demonstrates the potential of a Public Health Hackathon as an applied, experiential educational model for engaging future public health workforce in addressing contemporary public health challenges. By integrating the program within school contexts, students were provided with a structured platform to develop, test, and present campaigns focused on health issues relevant to adolescents, extending learning beyond the classroom.

The global, hybrid Hackathon was intentionally designed as a pragmatic, real-world learning experience rather than a controlled intervention, reflecting how public health ideas are developed and communicated in practice. Grounded in design thinking and applied public health education, the model prioritized accessibility, flexibility, and experiential learning.

A key contribution was the emphasis on skill development across the full project lifecycle, including problem identification, evidence review, human-centered design, project planning, prototyping, and communication. The program required no prior public health training, enabling students from diverse educational backgrounds to engage meaningfully in public health problem-solving. The Hackathon also functioned as a peer learning ecosystem, exposing participants to diverse project types and broadening their understanding of public health solutions. The design thinking-informed structure supported iterative learning, balancing creativity with feasibility and evidence.

While flexible mentorship and optional workshops enhanced scalability, participation levels varied, and outcomes were assessed primarily through project submissions rather than longitudinal measures. Future research should examine longer-term educational and workforce impacts.

Overall, the Hackathon illustrates the value of design-driven, experiential learning models in building public health capacity, creativity, and career awareness among future leaders, offering a scalable framework for engaging the future public health workforce.

### Implications for public health education and workforce development

The RE-AIM Public Health IDEAS Hackathon offers several implications for public health education and workforce development. First, it highlights the value of design-driven, experiential learning models that emphasize process-oriented skills—such as problem framing, ideation, prototyping, and communication—alongside foundational public health knowledge. These competencies are increasingly important for addressing complex, real-world public health challenges but are often underemphasized in traditional curricula.

Second, the Hackathon demonstrates how flexible, low-barrier programming can expand access to public health learning across diverse educational contexts. Optional workshops, asynchronous resources, and centralized mentorship enabled participation across varying institutional capacities, geographic locations, and student schedules, supporting scalability in hybrid and global settings.

Third, the model supports early career exploration and professional identity formation by exposing participants to diverse public health roles and solution pathways. Through peer learning and project visibility, participants gained a broader understanding of public health as a multidisciplinary field, informing future academic, professional, or civic engagement pathways.

Finally, by prioritizing practice-oriented learning over narrowly defined outputs, the Hackathon fostered a supportive environment for experimentation and iterative problem-solving. This approach aligns with workforce development priorities that emphasize adaptability, creativity, and applied problem-solving in an evolving public health landscape.

## Conclusion

This study describes the design and implementation of a global, hybrid public health hackathon that integrates future public health workforce engagement, design thinking, and applied public health education. Implemented as a pragmatic, real-world learning experience, the Hackathon emphasized accessibility, flexibility, and experiential skill building. Participants developed competencies across the public health project lifecycle, including problem identification, evidence use, human-centered design, prototyping, and communication.

Beyond individual skill development, the Hackathon functioned as a shared learning environment that exposed participants to diverse public health challenges and solution approaches, fostering creativity and broadening understanding of public health action and career pathways.

Although outcomes were assessed primarily through project submissions, the Hackathon demonstrates the potential of design-driven, youth-centered programming to complement traditional public health education and workforce development efforts. This adaptable model offers a scalable approach for schools and universities seeking to promote public health engagement, civic participation, and workforce readiness. Future work should examine longitudinal outcomes and adaptation across diverse educational and community contexts.

## Data Availability

The original contributions presented in the study are included in the article/Supplementary material, further inquiries can be directed to the corresponding author.

## Supplementary material

SUPPLEMENTARY FIGURE S1 Timeline showing key milestones of Public Health Hackathon.
