# Supplementary material for: Public Health Hackathon: empowering high school students as tomorrow’s leaders and innovators in public health
Source: Front Public Health. 2026 Feb 11;14:1745900. doi: 10.3389/fpubh.2026.1745900 (PMC12932556; doi:10.3389/fpubh.2026.1745900)
Supplement: SUPPLEMENTARY FIGURE S1 — Timeline showing key milestones of Public Health Hackathon. [file Supplementary_file_1.docx]

**Supplement Fig 1. Timeline showing key milestones of Public Health Hackathon**
