# Supplementary material for: Public Health Hackathon: empowering high school students as tomorrow’s leaders and innovators in public health
Source: Front Public Health. 2026 Feb 11;14:1745900. doi: 10.3389/fpubh.2026.1745900 (PMC12932556; doi:10.3389/fpubh.2026.1745900)
Supplement: Supplementary file 2 [file Table_1.docx]

**Supplemental Table 1. Hackathon Workshop Structure, Tools, and Expected Outputs**

| **Workshop** | **Focus** | **Key Tools & Frameworks** | **Expected Outputs** |
| --- | --- | --- | --- |
| Workshop 1 | Problem Identification & Research | FINER, PICOT, literature review, CDC/WHO data, stakeholder mapping | Defined problem statement; preliminary research |
| Workshop 2 | Ideation & Storyboarding | Brainstorming, empathy maps, HMW statements, dot voting, storyboards | Multiple concepts; selected solution |
| Workshop 3 | Prototyping & Project Design | Low-fidelity prototyping, wireframing, role/service prototyping | Prototype sketches; implementation plan |
| Workshop 4 | Pitching & Communication | CREATE criteria, elevator pitch, storytelling, visual design | Final abstract; recorded pitch |
