# Supplementary material for: Public Health Hackathon: empowering high school students as tomorrow’s leaders and innovators in public health
Source: Front Public Health. 2026 Feb 11;14:1745900. doi: 10.3389/fpubh.2026.1745900 (PMC12932556; doi:10.3389/fpubh.2026.1745900)
Supplement: Supplementary file 3 [file Table_2.docx]

**Supplemental Table 2. Abstract Evaluation Rubric (Maximum 50 Points)**

**Instructions for Reviewers:**
Reviewers evaluated abstracts aligned with the Hackathon theme *“Innovate for Health: Empower Youth for Digital Health and Well-Being”* and SDG 3 (*Ensure healthy lives and promote well-being for all ages*). Each criterion was scored from 1–10, for a total possible score of 50 points.

| **Criterion (10 points each)** | **Description** | **Key Considerations** |
| --- | --- | --- |
| **Problem Statement** | Evaluates how clearly the public health problem is defined and contextualized | Is the problem clearly articulated? Is relevant background provided? Does it align with youth and/or digital health and SDG 3? |
| **Solution / Innovation** | Assesses novelty, desirability, feasibility, and viability of the proposed solution | Is the solution innovative and distinct? Does it address a real public health need? Can it be realistically implemented? |
| **Approach** | Evaluates the clarity and structure of the proposed implementation plan | Are steps logical and actionable? Is the approach evidence-informed? Does it include youth and/or digital health components? |
| **Impact** | Assesses anticipated public health impact and evaluation plan | Are expected outcomes described? Are success indicators or evaluation measures included? |
| **Sustainability** | Evaluates long-term viability and potential for scale | Is there a plan for sustaining the project? Does it consider scalability or continued youth engagement? |

**Cross-cutting Evaluation Dimensions:**
Creative • Research-Oriented • Entrepreneurial • Accessible • Transformative • Effective for the Theme
