# Supplementary material for: Public Health Hackathon: empowering high school students as tomorrow’s leaders and innovators in public health
Source: Front Public Health. 2026 Feb 11;14:1745900. doi: 10.3389/fpubh.2026.1745900 (PMC12932556; doi:10.3389/fpubh.2026.1745900)
Supplement: Supplementary file 4 [file Table_3.docx]

**Supplemental Table 3. Pitch Evaluation Rubric (Maximum 80 Points)**

**Instructions for Judges:**
Judges scored each pitch on a scale of 1–10 for each category. Total possible score: 80 points.

| **Category** | **Description** |
| --- | --- |
| **Innovation & Creativity** | Assesses originality and uniqueness of the proposed solution relative to existing approaches |
| **Impact & Relevance** | Evaluates the significance of the public health issue addressed and potential for real-world impact |
| **Feasibility & Implementation** | Assesses practicality, clarity of implementation steps, and achievability with available resources |
| **Presentation & Clarity** | Evaluates organization, clarity of communication, and effectiveness of delivery |
| **Evidence & Data Support** | Assesses use of relevant data, research, or case examples to support the proposal |
| **Scalability & Sustainability** | Evaluates potential for expansion, adaptation, and long-term sustainability |
| **Team Collaboration & Passion** | Assesses teamwork, preparedness, enthusiasm, and commitment demonstrated during the pitch |
| **Visuals & Design** | Evaluates effectiveness of slides, graphics, prototypes, or other visual aids in supporting the pitch |

**Scoring Notes (Optional to Include Beneath Table)**

- Each category scored independently (1 = Needs Improvement, 10 = Outstanding).
- Judges could provide qualitative feedback to support learning and refinement.
- Final awards were determined using combined abstract and pitch scores, with separate consideration for high school and university teams.
