# Supplementary material for: Public Health Hackathon: empowering high school students as tomorrow’s leaders and innovators in public health
Source: Front Public Health. 2026 Feb 11;14:1745900. doi: 10.3389/fpubh.2026.1745900 (PMC12932556; doi:10.3389/fpubh.2026.1745900)
Supplement: Supplementary file 5 [file Table_4.docx]

**Supplemental Table 4**

**Summary of Student Projects Submitted to the 2024–2025 RE-AIM Hackathon**

| **Project Title** | **Institution** | **Country** | **Public Health Focus** |
| --- | --- | --- | --- |
| Cordova Stop STIs | Cordova High School (Seniors) | USA | Sexual health campaign and mobile clinic |
| ENGAGE | Panimalar Medical College Hospital & Research Institute | India | AI-powered childhood obesity prevention app |
| Kingsbury Optional Program | Kingsbury High School | USA | Substance abuse prevention for middle school students |
| Cordova CARES | Cordova High School (Juniors) | USA | School-based hygiene closet for equity |
| Southwind Smiles | Southwind High School | USA | Smart toothbrush and app for oral health |
| Malta Youth Wellness Initiative | De La Salle College Sixth Form School | Malta | Afterschool wellness program addressing obesity, vaping, addiction |
| Quit Smoking Accountability | Central High School | USA | Community smoking cessation program |
| HIV & AIDS Support & Education | Central High School | USA | Peer-led HIV awareness and stigma reduction |
| Green Seats | Musana Vocational High School (High School and College) | Uganda | Recycled plastic bottle seating for schools |
| Indohana | University of Memphis | USA | Cultural integration and mental health support app |
| GenSafe | Lincoln Community High School & University of Memphis | USA | Online safe spaces toolkit for youth |
| Alcohol Abuse Prevention I | Central High School | USA | Community-based alcohol reduction program |
| Alcohol Abuse Prevention II | Central High School | USA | Youth-focused alcohol awareness initiative |
| COVID Awareness | Central High School | USA | Post-pandemic health and safety awareness |
| Mental Health Support App | Central High School | USA | Mobile app for youth mental health |
| Substance Abuse Prevention | Central High School | USA | Education, treatment, and recovery support |

Public Health Hackathon topics submitted by the participants
